# Influence of Molecular Parameters on Rate Constants of Thermal Dissociation/Recombination Reactions: The Reaction System CF_4_ ⇄ CF_3_ + F

**DOI:** 10.1021/acs.jpca.3c00011

**Published:** 2023-02-13

**Authors:** Carlos J. Cobos, Elsa Tellbach, Lars Sölter, Jürgen Troe

**Affiliations:** † INIFTA, Facultad de Ciencias Exactas, Universidad Nacional de La Plata, CONICET, CP 1900 La Plata, Argentina; ‡ Max-Planck-Institut für Multidisziplinäre Naturwissenschaften, Am Fassberg 11, D-37077 Göttingen, Germany; § Institut für Physikalische Chemie, Universität Göttingen, Tammannstr. 6, D-37077 Göttingen, Germany

## Abstract

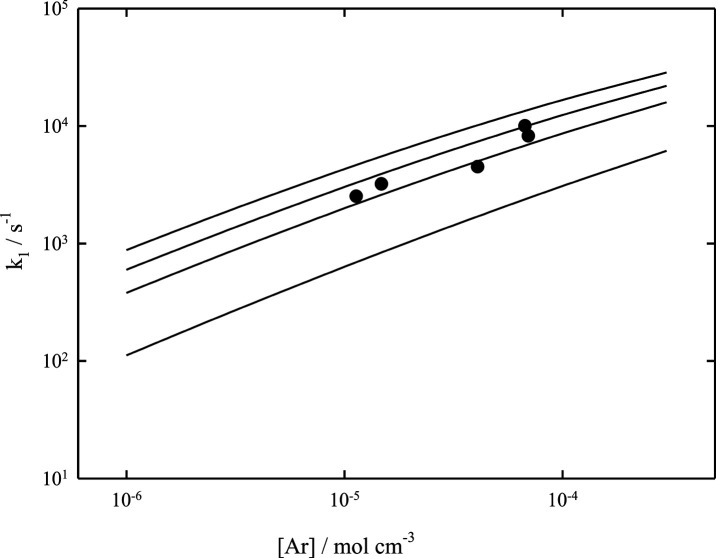

The possibilities to extract incompletely characterized molecular parameters from experimental thermal rate constants for dissociation and recombination reactions are explored. The reaction system CF_4_ (+M) ⇄ CF_3_ + F (+M) is chosen as a representative example. A set of falloff curves is constructed and compared with the available experimental database. Agreement is achieved by minor (unfortunately not separable) adjustments of reaction enthalpy and collisional energy transfer parameters.

## 1 Introduction

Statistical theories of unimolecular reactions have reached a mature state such that they can be used to predict rate constants of thermal dissociation/recombination reactions over a wide range of conditions (see, e.g., refs (1) and (2)). Obviously, this is of considerable practical importance. Nevertheless, there are often molecular parameters contributing to the rate constants that are not well known and that require detailed considerations, either by theoretical modeling or by educated guessing. In the following, we demonstrate the influence of selected molecular parameters on the rate constants. Such parameters are the average energy ⟨Δ*E*⟩_total_ transferred per collision between the excited molecules and the surrounding bath gas species, characteristic parameters of the potential energy surface (PES) of the reaction system, and, for dissociation reactions in particular, the enthalpy Δ*H*^o^ of the reaction. These quantities all influence the rate constants, but they do it in different ways. It may not be easy to unravel their respective contributions. This is the issue of the present article.

The dissociation reaction
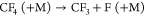
1and the reverse recombination reaction
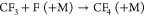
2are chosen as representative examples (where M stands for an arbitrary partner for collisional energy transfer). The experimental database is limited (for the dissociation reaction 1, see refs (3)–^5^; for the recombination reaction 2, see refs (6)–^8^). We illustrate the influence of the mentioned molecular parameters on the respective rate constants *k*_1_ and *k*_2_, and we try to specify their values by combining experimental and modeling results. Regardless of the precision of the derived parameters, the described approach allows one to construct an internally consistent set of falloff curves and to identify uncertainties of the analysis. In this way, it may help improve recommendations of rate constants (see, e.g., the difference between various experimental results reported in ref (8)).

## 2 Modeling Approach

The falloff curves of the rate constants *k*_1_ and *k*_2_ are dominated by limiting low-pressure (subscript 0) and high-pressure (subscript ∞) rate constants. For this reason, attention is first focused on these limiting rate constants. Approximate expressions for the transition of *k*_1_ from *k*_1,0_ to *k*_1,∞_ (or *k*_2_ from *k*_2,0_ to *k*_2,∞_) are available (see, e.g., refs (9)–^11^) and are used when the limiting rate constants have been fixed. Here, we employ the expressions recommended in ref (10), see below.

High-pressure recombination rate constants *k*_2,∞_ correspond to rate constants *k*_cap_ for capture between the combining species. As long as the anisotropy of the PES can be neglected, *k*_cap_ is given by phase space theory (PST). For Morse-type PESs, classical trajectory and statistical adiabatic channel calculations in refs (12) and (13) lead to approximate analytical results for *k*_cap_^PST^, which are easily evaluated and show the influence of the Morse parameters β_e,_*r*_e_, and *D*_e_ in a transparent way. Defining *Y*(*X*) = *k*_cap_^PST^ (8π*kT*/μ)^−1/2^ β_e_^2^ and *X* = ln(*kT*/*D*_e_) – β_e_*r*_e_, one obtains
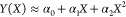
3with the parameters α_0_ = −15.7706, α_1_ = −8.6364, and α_2_ = 0.9975. If recombination from a single electronic level of the reactants into a single electronic state of the adduct is considered, the electronic weight factor *f*_el_ (given by the relevant ratio of electronic partition functions) has to be multiplied with *k*_cap_^PST^ in addition. Table 1 shows results for *k*_cap_^PST^ = *k*_2,∞_^PST^ as a function of the temperature *T*.

**Table 1:** Modeled Limiting High-pressure Rate Constants for the Dissociation Reaction CF_4_ → CF_3_ + F (*k*_1,∞_) and the Recombination Reaction CF_3_ + F → CF_4_ (*k*_2,∞_)^a^

*T*/K	*K*_eq_	*k*_2,∞_^PST^	*f*_rigid_	*k*_2,∞_	*k*_1,∞_
300		6.76 × 10^13^	1.92 × 10^–1^	1.30 × 10^13^	
1000	1.31 × 10^–25^	7.90 × 10^13^	1.74 × 10^–1^	1.37 × 10^13^	1.80 × 10^–12^
2000	1.38 × 10^–11^	8.70 × 10^13^	1.63 × 10^–1^	1.42 × 10^13^	1.95 × 10^2^
2500	7.81 × 10^–9^	8.99 × 10^13^	1.59 × 10^–1^	1.43 × 10^13^	1.12 × 10^5^
3000	5.15 × 10^–7^	9.23 × 10^13^	1.57 × 10^–1^	1.45 × 10^13^	7.44 × 10^6^
4000	8.69 × 10^–5^	9.62 × 10^13^	1.52 × 10^–1^	.46 × 10^13^	1.27 × 10^9^

a *k*_1,∞_ in s^-1^, *k*_2,∞_ in cm^3^mol^-1^ s^-1^, and equilibrium constant *K*_eq_ = *k*_1,∞_/*k*_2,∞_ in mol cm^-^^3^; superscript PST: phase space theory; rigidity factor *f*_rigid_ = *k*_2,∞_/*k*_2,∞_^PST^, see the text.

Accounting for the anisotropy of the PES reduces *k*_2,∞_ to values below *k*_2,∞_^PST^. This is accounted for by a rigidity factor *f*_rigid_, which is smaller than unity, that is,
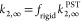
4

If the anisotropy of the PES is represented by an exponential decay of the frequency ε(*r*) [or the frequencies ε_*i*_(*r*)] of transitional modes along the length *r* of the forming bond {of the form ε(*r*) ≈ ε(*r*_e_) exp[−α_e_ (*r* – *r*_e_)]} and if α_e_ is of the order of 0.5 β_e_, according to ref (12), *f*_rigid_ can be approximated by
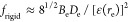
5Eq 5 provides a quick access to *f*_rigid_ and shows its dependence on molecular parameters (*B*_e_ here denotes the relevant rotational constant of the forming adduct, see below). It should be noted that the derivation of eq 5 in ref (12) was based on the validity of an approximate relation
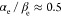
6between α_e_ and β_e_. Deviations from eq 5 of less than about ±10% were observed when α_e_/β_e_ differed from 0.5 by less than ±10%. Anisotropies characterized by eq 6 initially were suggested by the analysis of experimental results, see ref (14). Quantum chemical calculations, meanwhile, often confirmed the validity of this relationship (see also the results for the present reaction system shown in the Supporting Information). While the dependence of *k*_2,∞_ on details of the PES is only weak, this is different for the limiting high-pressure rate constant *k*_1,∞_. As *k*_1,∞_ follows from *k*_2,∞_ through the equilibrium constant *K*_eq_ = *k*_1,∞_/*k*_2,∞_ and as *K*_eq_ and *k*_1,∞_ both contain the strongly temperature-dependent Boltzmann factor exp(−Δ*H*_0_^o^/*kT*), the analysis of *k*_1,∞_ can be used to determine Δ*H*_0_^o^. Table 1 compares calculated temperature dependences of *k*_1,∞_ and *k*_2,∞_, *k*_1,∞_^PST^ and *k*_2,∞_^PST^, as well as the corresponding thermal rigidity factors *f*_rigid_ (the used molecular parameters of the calculations are given in the Supporting Information).

The calculation of limiting low-pressure rate constants *k*_1,0_ and *k*_2,0_ has been described in detail in ref (11) and is not reproduced here. *k*_1,0_ has been expressed in the form
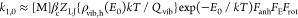
7with various contributions characterized by individual factors. Table 2 shows calculations of such factors (the critical energy *E*_0_ here is identified with Δ*H*_0_^o^; temperature-dependent quantities are given for 300 K. While the calculation of most of the factors is straightforward and shows the dependence on molecular parameters in a transparent manner, the rotational factor *F*_rot_ requires attention. It depends on the centrifugal barriers *E*_0_(*J*) of the system and, hence, on properties of the PES like the rotational constant of the system along the dissociating bond (see the Supporting Information). Properties of collisional energy transfer enter through the overall Lennard-Jones collision frequency *Z*_LJ_ and a collision efficiency β_c_. Information on the latter quantity can be derived from the analysis of experimental values of *k*_1,0_ and *k*_2,0_ and is of particular importance. Through the solution of simplified master equations, a relation between β_c_ and the average (total) energy ⟨Δ*E*⟩_total_ of the form
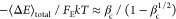
8was derived (see refs (11) and (15); *F*_E_ corrects for the used expression of the energy dependence of the vibrational density of states ρ_vib,h_(*E*); *F*_E_ in general is not far from unity; it should be noted that ⟨Δ*E*⟩_total_ differs from the average energy transferred per down-transition, ⟨Δ*E*⟩_down_, and the two quantities are related by ⟨Δ*E*⟩_total_ ≈ −⟨Δ*E*⟩_down_^2^/(⟨Δ*E*⟩_down_ + *F*_E_*kT*) such that β_c_ ≈ [⟨Δ*E*⟩_down_/(⟨Δ*E*⟩_down_ + *F*_E_*kT*)]^2^, see ref (15)). Table 2 includes values of β_c_ estimated with a value of −⟨Δ*E*⟩_total_/*hc* = 100 cm^–1^; later on, ⟨Δ*E*⟩_total_ will be used as an empirical fit parameter to be extracted from those experiments that are sensitive to *k*_1,0_ and *k*_2,0_.

**Table 2:** Contributing Quantities to Equation 7 for Limiting Low-pressure Rate Constants of the Recombination Reaction CF_3_ + F (+He) → CF_4_ (+He) at 300 K [^a^in cm^3^ mol^–1^ s^–1^, ^b^in (kJ mol^–1^)^−1^, ^c^Collision Efficiency β_c_ Calculated with −⟨Δ*E*⟩_total_/*hc* = 100 cm^–1^; ^d^in cm^6^ mol^–2^ s^–1^; Equation 7 from Reference (11)]

quantity in eq 7	value
*Z*_LJ_^a^	2.58 × 10^14^
*Q*_vib_	1.54
ρ_vib,h_(*E*_0_)^b^	7.54 × 10^8^
*F*_anh_	1.79
*F*_E_	1.03
*F*_rot_	15.5
β_c_^c^	0.24
*k*_rec,0_/[He]^d^	1.54 × 10^21^

Falloff curves connecting the limiting low- and high-pressure rate constants primarily are expressed in the Lindemann–Hinshelwood form; however, additionally accounting for a broadening of the falloff curves
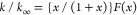
9where *x* = *k*_1,0_/*k*_1,∞_ or *x* = *k*_2,0_/*k*_2,∞_ are proportional to [M] such that *x* represents a reduced pressure scale. Broadening factors *F*(*x*) (being smaller than unity) here are approximated in the form recommended in refs (9)–^11^. The deviations of the falloff curves from the Lindemann–Hinshelwood form are most pronounced near the center of the curves, that is, close to those values of [M] where *k*_1,0_ is equal to *k*_1,∞_ (or where *k*_2,0_ is equal to *k*_2,∞_). The corresponding center broadening factors *F*_cent_ can be estimated using the methods outlined in refs (9)–^11^ and (16). They can be related to effective numbers of oscillators of the system, but they also contain energy transfer contributions. Symmetric broadening factors *F*(*x*) = *F*(1/*x*) of the form
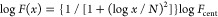
10with *N* = 0.75–1.27 log *F*_cent_ from ref (11) often are sufficient for applications, but refined asymmetric expressions
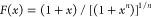
11with *n* = [ln 2/ln(2/*F*_cent_)] [0.8 + 0.2*x*^*q*^] and *q* = (*F*_cent_ – 1)/ln(*F*_cent_/10) have also been tested (see refs (9) and (10)).

*F*_cent_ has strong collision (for β_c_ = 1) and weak collision (β_c_ < 1) contributions, that is, *F*_cent_ = *F*_cent_^sc^*F*_cent_^wc^, with *F*_cent_^sc^ as given above (from refs (9)–^11^ and (16)) and *F*_cent_^wc^ = max (β_c_^0.14^, 0.64) from ref (9). *F*_cent_ first increases with temperature, and then, it decreases again. Representative values for the present system (with M = Ar) are *F*_cent_ ≈ 0.74, 0.17, 0.095, 0.084, and 0.089 for *T*/K = 300, 1000, 2000, 3000, and 4000, respectively. Obviously, the values of the limiting rate constants are more important for the construction of the falloff curves than the precise values of *F*_cent_. Table 3 summarizes the derived values of *k*_1,0_, *k*_1,∞_, *k*_2,0_, and *k*_2,∞_, together with the corresponding *K*_eq_ and approximate values for *F*_cent_.

**Table 3:** Modeled Limiting High-pressure (Subscript ∞) and Low-pressure (Subscript 0) Rate Constants for the Dissociation Reaction CF_4_ (+Ar) → CF_3_ + F (+Ar) (*k*_1_, for Temperatures 1000–4000 K) and the Recombination Reaction CF_3_ + F (+Ar) → CF_4_ (+Ar) (*k*_2_, for Temperatures 300–1000 K)^a^

*k*_1,∞_ = 4.0 × 10^16^ (*T*/2500 K)^−1.4^ exp(−66,500 K/*T*) s^–1^	1000–4000 K
*k*_2,∞_ = 1.4 × 10^13^ (*T*/800 K)^0.04^ cm^3^ mol^–1^ s^–1^	300–1000 K
*k*_1,0_/[Ar] = 7.8 × 10^20^ (*T*/2500 K)^−9.6^ exp(−70,000 K/*T*) cm^3^ mol^–1^ s^–1^	1000–4000 K
*k*_2,0_/[Ar] = 5.4 × 10^20^ (*T*/800 K)^−5.4^ exp(−1160 K/*T*) cm^6^ mol^–2^ s^–1^	300–1000 K
*F*_cent_ ≈ exp(−*T*/155 K) + exp(−*T*/600 K) + exp(−4880 K/*T*)	300–4000 K

a Energy transfer was characterized by −⟨Δ*E*⟩_total_/*hc* = 500 cm^–1^. Modeled center broadening factors *F*_cent_ of 0.75, 0.57, 0.39, 0.26, 0.21, and 0.12 at *T* = 300, 400, 600, 800, 1000, and 2000 K, respectively, were approximated by the given expression.

## 3 Evaluation of Experimental Results

**Figure 1:**
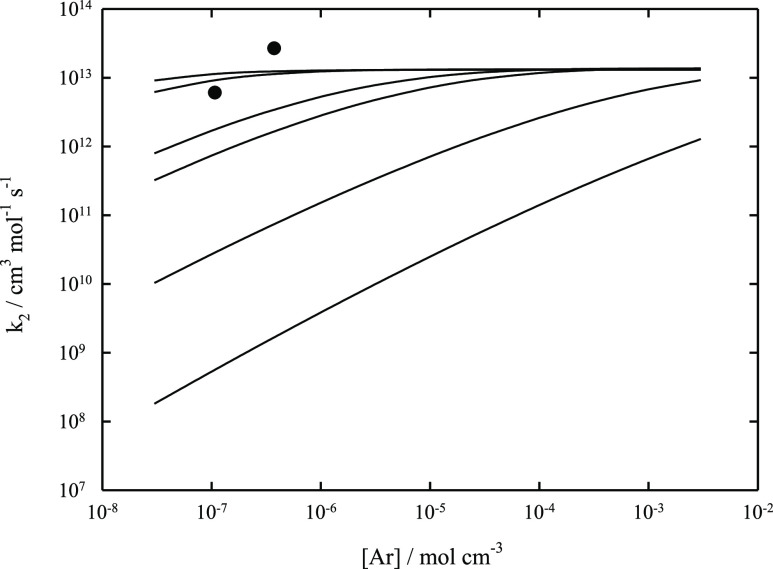
Falloff curves for the recombination reaction CF_3_ + F (+Ar) → CF_4_ (+Ar) at *T* = 300, 400, 800, 1000, 2000, and 4000 K (from top to bottom; modeled curves for −⟨Δ*E*⟩_total_/*hc* = 500 cm^–1^; experimental points from ref (7)).

**Figure 2:**
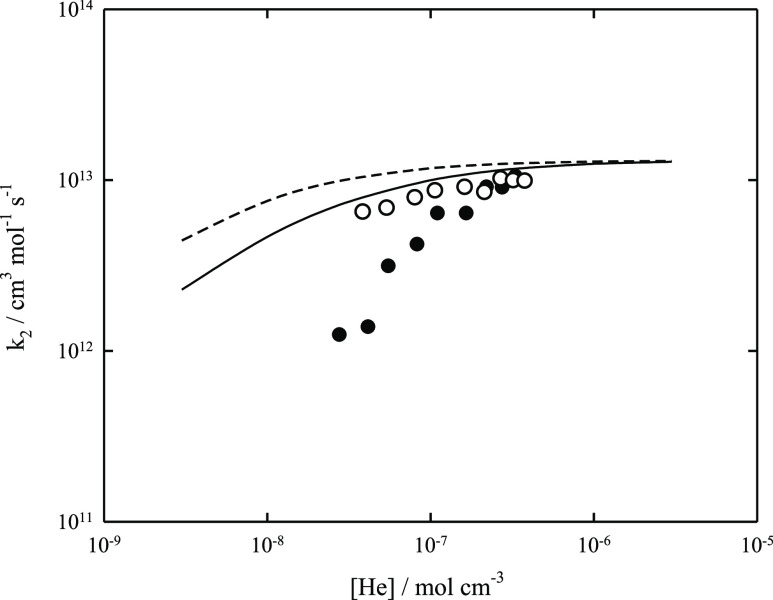
Falloff curve for the recombination reaction CF_3_ + F (+He) → CF_4_ (+He) at *T* = 300 K. Solid line: modeled curve using *k*_2,∞_ = 1.30 × 10^13^ cm^3^ mol^–1^ s^–1^, *k*_2,0_/[He] = 1.54 × 10^21^ cm^6^ mol^–2^ s^–1^ (calculated with −⟨Δ*E*⟩_total_/*hc* = 100 cm^–1^), and *F*_cent_ = 0.66. Dashed line: modeled curve using *k*_2,∞_ = 1.30 × 10^13^ cm^3^ mol^–1^ s^–1^, *k*_2,0_/[He] = 3.70 × 10^21^ cm^6^ mol^–2^ s^–1^ (calculated with −⟨Δ*E*⟩_total_/*hc* = 500 cm^–1^), and *F*_cent_ = 0.74. Experimental points: ○ from ref (6) and ● from ref (8).

The comparison of experimental results with modeled falloff curves such as that described in Section 2, on one hand, validates the used molecular parameters of the system entering the given analysis. On the other hand, it allows one to locate the experimental rate constants at their proper position along the falloff curves. We illustrate this with the available experimental data for reactions 1 and 2. The rate constant *k*_2_ in the bath gas Ar was first measured in ref (7) at room temperature and at [Ar] = 1.1 × 10^–7^ and 3.7 × 10^–7^ mol cm^–3^. As an increase of the measured *k*_2_ by about a factor of 4 has been observed, the recombination reaction was assumed to be near its third-order range. The comparison of the data with modeled falloff curves from the present work in Figure 1, however, casts doubt on this interpretation of the measurements. Nevertheless, the measured absolute value of *k*_2_ = 2.6 (±0.7) × 10^13^ cm^3^ mol^–1^ s^–1^ at [Ar] = 3.7 × 10^–8^ mol cm^–3^ appears roughly reconcilable with the present calculated value of *k*_2,∞_ = 1.3 × 10^13^ cm^3^ mol^–1^ s^–1^. Meanwhile, the modeling predicts a weaker dependence on [M] than that claimed for the experiments. Indeed, the later measurements of refs (6) and (8) (in He between 3.9 × 10^–8^ and 3.8 × 10^–7^ mol cm^–3^) showed weaker pressure dependences than that found in ref (7). Figure 2 compares the corresponding results with a modeled falloff curve for M = He. The remaining differences between the results from refs (6) and (8) cannot be explained by uncertainties in the used molecular parameters, in particular in the used value for ⟨Δ*E*⟩_total_. Therefore, the modeled falloff curves of Figures 1 and 2 based on assumed values of −⟨Δ*E*⟩_total_/*hc* = 100 or of 500 cm^–1^ for M = He or of Ar appear to provide a more reasonable representation of the pressure dependence of *k*_2_. The agreement with the experiments from ref (6) appears quite satisfactory, while the difference from the results from ref (8) calls for a reinterpretation of the assumed mechanism of the experiments.

**Figure 3:**
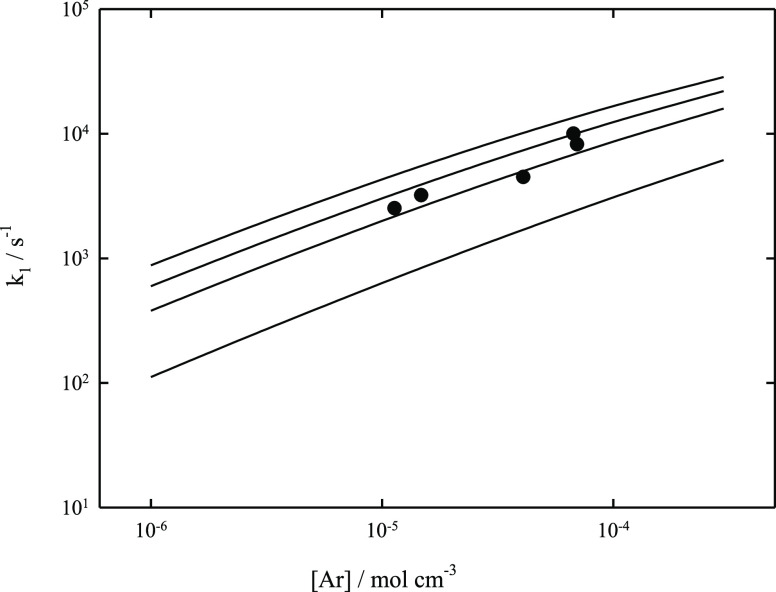
Falloff curves for the dissociation reaction CF_4_ (+Ar) → CF_3_ + F (+Ar) at *T* = 2500 K (modeled curves for −⟨Δ*E*⟩_total_/*hc* = 100, 500, 1000, and 2000 cm^–1^ from bottom to top, modeling with rate constants *k*_1_,_0_ and *k*_1_,_∞_ as well as *F*_cent_ from Table 3; ●: experimental points from ref (4)).

**Figure 4:**
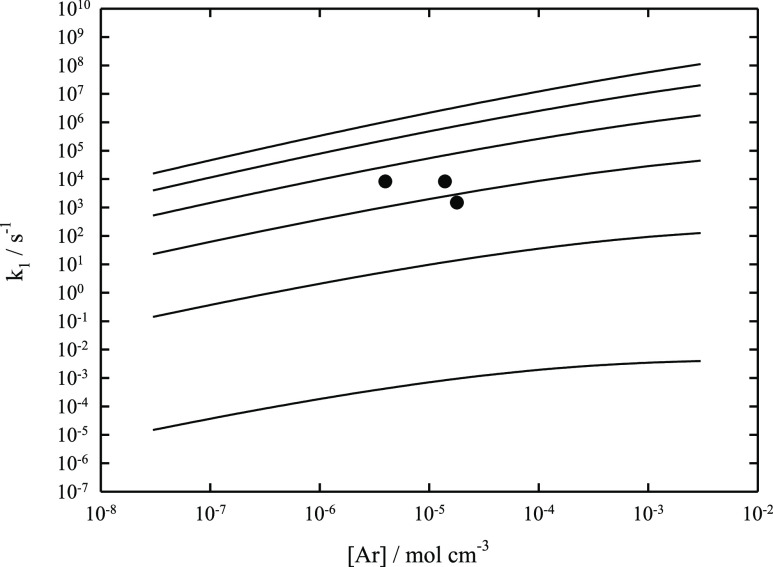
Modeled falloff curves for the dissociation reaction CF_4_ (+Ar) → CF_3_ + F (+Ar) at the temperatures *T* = 1500, 2000, 2500, 3000, 3500, and 4000 K (from bottom to top; modeling with rate constants *k*_1,0_ and *k*_1,∞_ as well as *F*_cent_ from Table 3 and for −⟨Δ*E*⟩_total_/*hc* = 500 cm^–1^; ●: representative experimental points from ref (3) at temperatures between 2400 and 3000 K).

The question arises how well measured dissociation rate constants *k*_1_ agree with modeling results and whether they provide a further access to molecular parameters. Figure 3 compares measurements of *k*_1_ at *T* = 2500 K and [Ar] between 10^–5^ and 10^–4^ mol cm^–3^ with modeling results for a series of values of −⟨Δ*E*⟩_total_/*hc*. The best agreement seems to be obtained with a value of −⟨Δ*E*⟩_total_/*hc* of the order of 500 cm^–1^. Modeling of the temperature dependence of the corresponding falloff curves in Figure 4 clearly confirms that the dissociation measurements have been made far below the high pressure limit of the reaction, while recombination measurements at 300 K were made close to the high pressure limit of the reaction. As the latter thus practically cannot provide an access to ⟨Δ*E*⟩_total_, one might be tempted to extract ⟨Δ*E*⟩_total_ from Figure 3. However, there is an alternative interpretation. Besides ⟨Δ*E*⟩_total_, *k*_1_ in Figure 3 also depends on the precise value of the reaction enthalpy Δ*H*_0_^o^. Decreasing −⟨Δ*E*⟩_total_/*hc* from 500 to 100 cm^–1^ in Figure 3 would decrease the modeled *k*_1_ at [Ar] = 10^–5^ mol cm^–3^ by about a factor of 2.9. This decrease could be compensated by a decrease of Δ*H*_0_^o^ of reaction 1 by about 22 kJ mol^–1^. As the uncertainty of Δ*H*_0_^o^ apparently is markedly smaller today (see the Supporting Information), it appears more probable that −⟨Δ*E*⟩_total_/*hc* in the present case is of the order of 500 cm^–1^, such as that fitted in Figure 3, although this value is larger than the value of 100 cm^–1^ generally assumed in our modeling. In any case, this example illustrates the sensitivity of the modeled rate constants on molecular parameters. Unfortunately, the thermochemical parameter Δ*H*_0_^o^ and the energy transfer parameter ⟨Δ*E*⟩_total_ cannot be separated in the analysis. In particular, no conclusion on the temperature dependence of ⟨Δ*E*⟩_total_ for the reaction CF_4_ (+Ar) ⇄ CF_3_ + F (+Ar) can be drawn. In other cases [e.g., the CH_4_ (+Ar) ⇄ CH_3_ + H (+Ar) system analyzed in ref (17)], only a weak temperature dependence of ⟨Δ*E*⟩_total_ was postulated.

## 4 Conclusions

Regardless of whether correct molecular parameters have been used in the modeling or not, fixing ⟨Δ*E*⟩_total_ and Δ*H*_0_^o^ to the values used for the interpretation of Figure 3 and assuming only a weak temperature dependence of ⟨Δ*E*⟩_total_, the corresponding set of modeled falloff curves shown in Figure 4 provides an internally consistent picture of the temperature and pressure dependence of reactions 1 and 2. The outlined procedure illustrates the influence of various molecular parameters on the rate constants, and it may be used to identify problems of the interpretation of experimental results.

## Supporting Information Available

Molecular details of the reaction CF_4_ (+M) ⇄ CF_3_ + F (+M) (PDF)
