# Supplementary material for: Influence of Molecular Parameters on Rate Constants of Thermal Dissociation/Recombination Reactions: The Reaction System CF4 ⇄ CF3 + F
Source: J Phys Chem A. 2023 Feb 13;127(7):1697–701. doi: 10.1021/acs.jpca.3c00011 (PMC9969511; doi:10.1021/acs.jpca.3c00011)
Supplement: Supplementary file 1 — jp3c00011_si_001.pdf [file jp3c00011_si_001.pdf]

# Influence of Molecular Parameters on Rate Constants of Thermal Dissociation/Recombination Reactions: the Reaction System $CF_4 \rightleftarrows CF_3 + F$

Carlos J. Cobos [a], Elsa Tellbach [b,c], Lars Sölter[b,c],

and Jürgen Troe *[b,c]

Supporting Information

[a] INIFTA, Facultad de Ciencias Exactas, Universidad Nacional de La Plata, CONICET, La Plata, Argentina

[b] Max-Planck-Institut für Multidisziplinäre Naturwissenschaften, Am Fassberg 11, D- 37077 Göttingen, Germany,

[c] Institut für Physikalische Chemie, Universität Göttingen, Tammannstr. 6, D-37077 Göttingen, Germany

*Email:juergen.troe@mpinat.mpg.de

## SI –Molecular details of the reaction $CF_4$ (+ M) ⇄ $CF_3$ + F (+ M)

Quantum-chemical calculations of details of the potential energy surface were performed at the G4 ab initio composite level (ref SI1) with molecular structures and harmonic vibrational frequencies derived at the B3LYP/6-311+G(3df) DFT level of theory (ref SI2). The calculations were performed using the Gaussian 09 suite of programs with default integration grid (ref SI3).

## Details of the potential energy surface

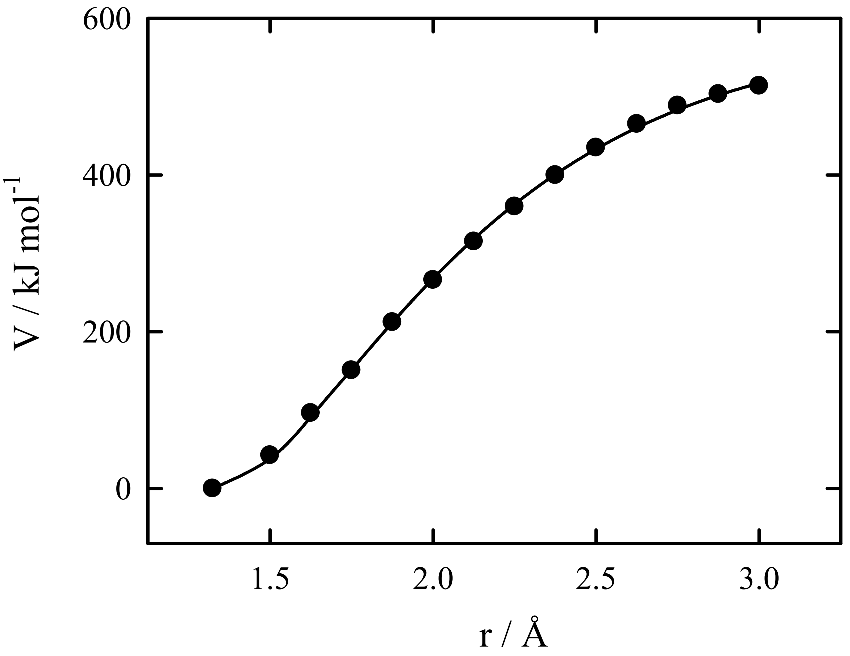


Fig. SI1 Electronic potential for the dissociation $CF_4 \rightarrow CF_3$ + F along the minimum energy path (MEP). G4//B3LYP/6-311+G(3df) calculations fitted with a Morse function with $D_e$ = 587.0 kJ mol$^{-1}$, $\beta_e$ = 1.66 Å$^{-1}$, and $r_e$ = 1.324 Å.

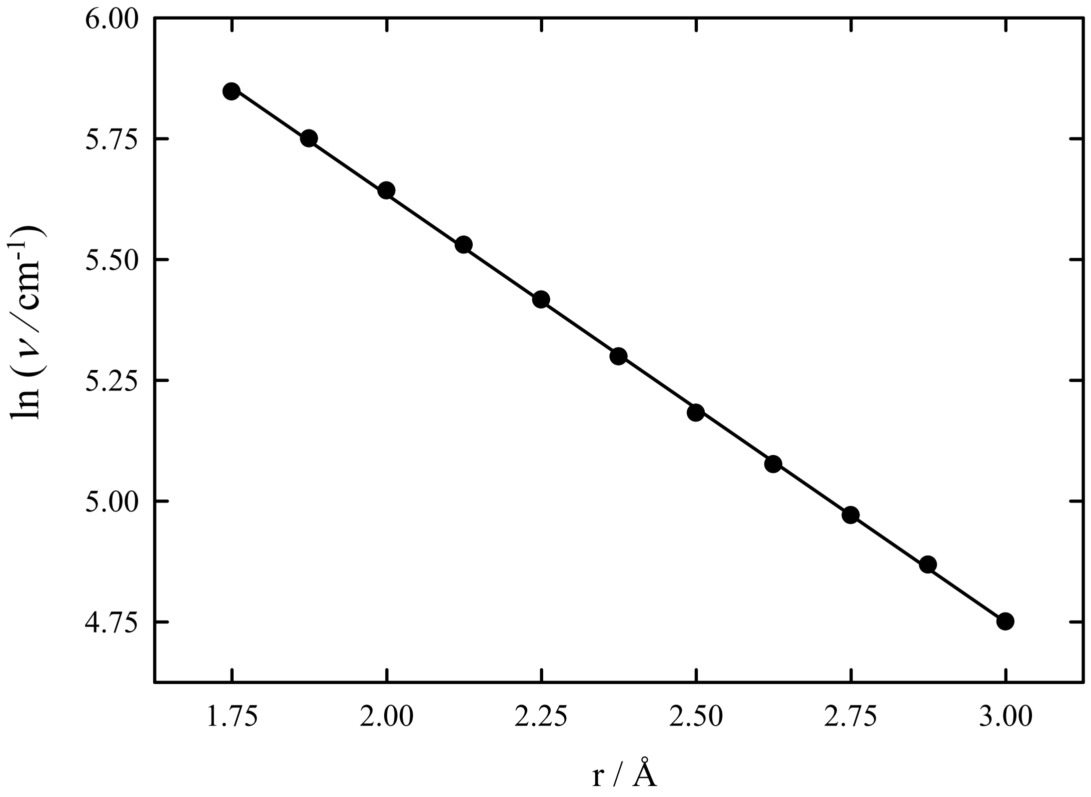


Fig. SI2 Transitional mode frequency (degenerate) for the dissociation $CF_4 \rightarrow CF_3 + F$ along the MEP, calculated at the B3LYP/6-311+G(3df) level of theory. The fitted decay parameter is equal to $\alpha_e = 0.89$ Å$^{-1}$.

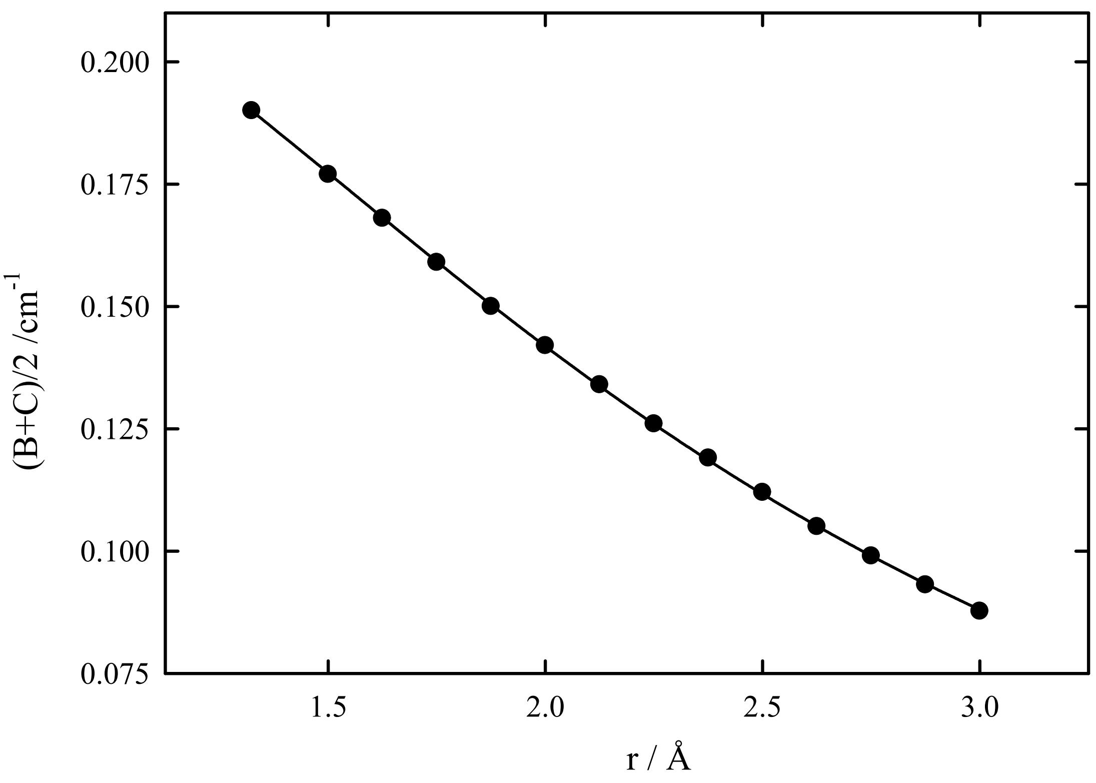


Fig. SI3 Rotational constants of $CF_4$ along the MEP of the dissociation $CF_4 \rightarrow CF_3 + F$, calculated at the B3LYP/6-311+G(3df) level of theory; results fitted with a function (B+C)/2 $cm^{-1}$ = $0.190/[1 + 0.3740(r - r_e) + 0.1895(r - r_e)^2]$ ($r$ in Å, $r_e$ = 1.324 Å).

## Calculation of rate constants for $CF_4$ dissociation

### High pressure range

| $T$ / K | $K_{eq}$ | $k_{rec,\infty}^{PST}$ | $f_{rigid}$ | $k_{1,\infty}$ | $k_{2,\infty}$ |
|---|---|---|---|---|---|
| 300 | $2.96 \times 10^{-94}$ | $6.76 \times 10^{13}$ | $1.92 \times 10^{-1}$ | $1.30 \times 10^{13}$ | $3.72 \times 10^{-81}$ |
| 1000 | $1.31 \times 10^{-25}$ | $7.90 \times 10^{13}$ | $1.74 \times 10^{-1}$ | $1.37 \times 10^{13}$ | $1.80 \times 10^{-12}$ |
| 1500 | $3.14 \times 10^{-16}$ | $8.34 \times 10^{13}$ | $1.67 \times 10^{-1}$ | $1.39 \times 10^{13}$ | $4.39 \times 10^{-3}$ |
| 2000 | $1.38 \times 10^{-11}$ | $8.70 \times 10^{13}$ | $1.63 \times 10^{-1}$ | $1.42 \times 10^{13}$ | $1.95 \times 10^{2}$ |
| 2500 | $7.81 \times 10^{-9}$ | $8.99 \times 10^{13}$ | $1.59 \times 10^{-1}$ | $1.43 \times 10^{13}$ | $1.12 \times 10^{5}$ |
| 3000 | $5.15 \times 10^{-7}$ | $9.23 \times 10^{13}$ | $1.57 \times 10^{-1}$ | $1.45 \times 10^{13}$ | $7.44 \times 10^{6}$ |
| 3500 | $9.76 \times 10^{-6}$ | $9.44 \times 10^{13}$ | $1.54 \times 10^{-1}$ | $1.45 \times 10^{13}$ | $1.42 \times 10^{8}$ |
| 4000 | $8.69 \times 10^{-5}$ | $9.62 \times 10^{13}$ | $1.52 \times 10^{-1}$ | $1.46 \times 10^{13}$ | $1.27 \times 10^{9}$ |

Table SI1. Equilibrium constants $K_{eq}$ (in mol cm$^{-3}$) and limiting high-pressure rate constants for the reactions $CF_3 + F \rightarrow CF_4$ (in cm$^3$ mol$^{-1}$ s$^{-1}$) and $CF_4 \rightarrow CF_3 + F$ (in s$^{-1}$) calculated with the parameters $\alpha_e$ and $\beta_e$ of Figs. SI1 and SI2 (i. e. with $\alpha_e / \beta_e$ = 0.89 Å$^{-1}$ / 1.66 Å$^{-1}$ = 0.54) and the rotational constants of Fig. SI3. SACM/CT calculations for $k_{2,\infty}$ in comparison to phase space theory (PST) with rigidity factors $f_{rigid} = k_{2,\infty} / k_{2,\infty}^{PST}$.

**Low pressure range**

| $T$/ K | $k_{1,0}$/[Ar]$^{SC}$ | $\beta_c$ | $k_{1,0}$/[Ar] |
|---|---|---|---|
| 300 | $2.59x10^{-70}$ | 0.569 | $1.47x10^{-70}$ |
| 1000 | $2.26x10^{-5}$ | 0.293 | $6.62x10^{-6}$ |
| 1500 | $9.17x10^{3}$ | 0.214 | $1.96x10^{3}$ |
| 2000 | $9.43x10^{7}$ | 0.167 | $1.57x10^{7}$ |
| 2500 | $1.61x10^{10}$ | 0.133 | $2.14x10^{9}$ |
| 3000 | $3.81x10^{11}$ | 0.109 | $4.15x10^{10}$ |
| 3500 | $3.02x10^{12}$ | 0.0905 | $2.73x10^{11}$ |
| 4000 | $1.24x10^{13}$ | 0.0760 | $9.42x10^{11}$ |

Table SI2. Limiting low-pressure rate constants $k_{1,0}$/[Ar] for $CF_4$ (+ Ar) $\rightarrow$ $CF_3$ + F (+ Ar) (in $cm^3$ $mol^{-1}$ $s^{-1}$); $F_{rot}$ calculated with the potential of Fig. SI1 and rotational constants of Fig.SI3. $-\langle\Delta E\rangle_{total}/hc$ = 500 $cm^{-1}$ was used. Calculations for strong collisions ($k_{1,0}$/[Ar]$^{SC}$); collision efficiencies $\beta_c = k_{1,0}/k_{1,0}^{SC}$.

**Falloff curves**

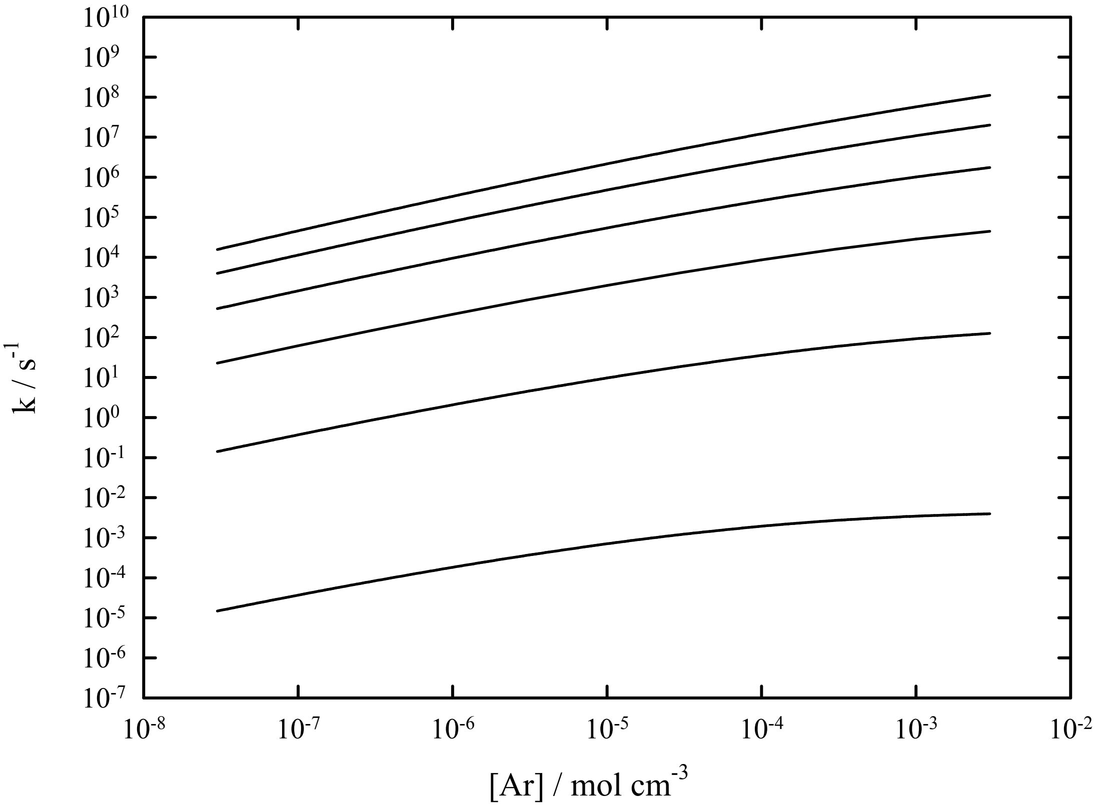


Fig. SI4 Modeled rate constants for the reaction $CF_4$ (+ Ar) $\rightarrow$ $CF_3$ + F (+ Ar) at $T$ = 1500, 2000, 2500, 3000, 3500, and 4000 K (from bottom to top) using $-\langle\Delta E\rangle_{total}/hc$ = 500 $cm^{-1}$, and center broadening factors $F_{cent}$ = 0.20 (1000 K), 0.14 (1500 K), 0.11 (2000 K), 0.11 (2500 K), 0.10 (3000 K), 0.10 (3500 K) and 0.11 (4000 K).

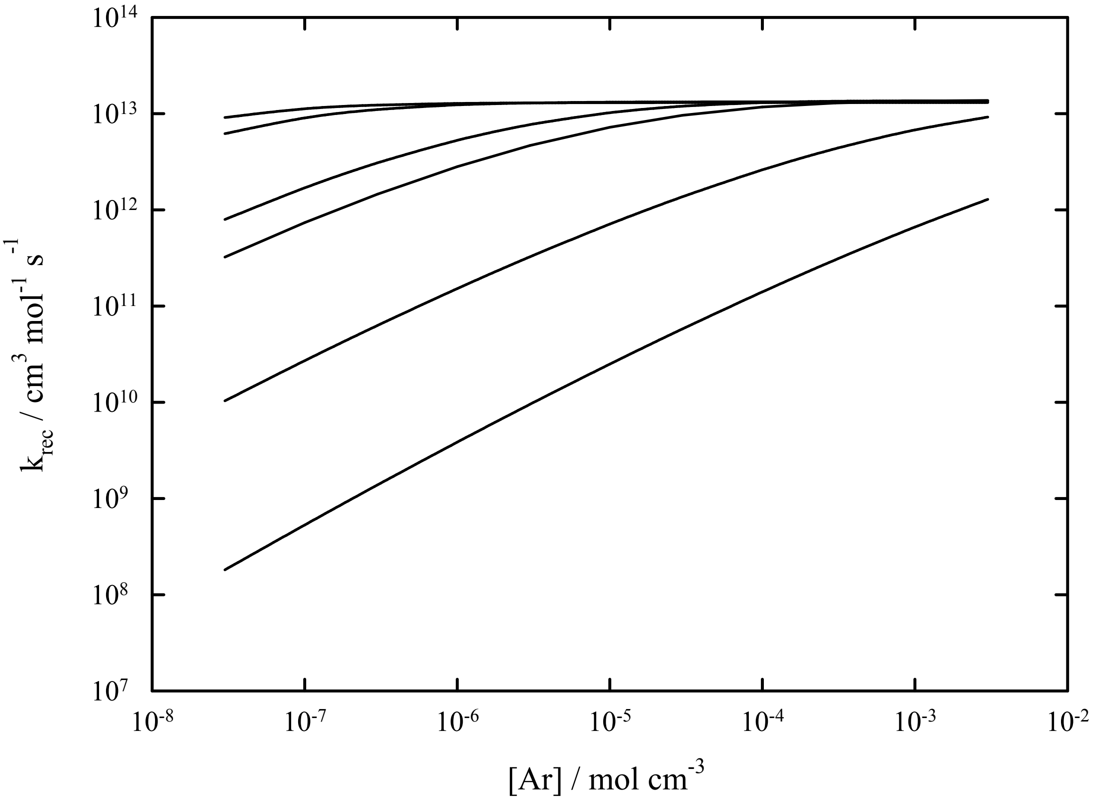


Fig. SI5. Modeled rate constants for the reaction $CF_3$ + F (+ Ar) → $CF_4$ (+ Ar) at for collisional energy transfer = 300, 400, 800, 1000, 2000, and 4000 K (from top to bottom) using $-\langle\Delta E\rangle_{total}/hc$ = 500 $cm^{-1}$. The used limiting rate constants were $k_{2,\infty}$ = $1.30\text{x}10^{13}$ (300 K), $1.33\text{x}10^{13}$ (400 K), $1.36\text{x}10^{13}$ (800 K), $1.37\text{x}10^{13}$ (1000 K), $1.41\text{x}10^{13}$ (2000 K) and $1.46\text{x}10^{13}$ $cm^3$ $mol^{-1}$ $s^{-1}$ (4000 K); $k_{2,0}$/[Ar] = $2.33\text{x}10^{21}$ (300 K), $1.27\text{x}10^{21}$ (400 K), $1.29\text{x}10^{20}$ (800 K), $5.04\text{x}10^{19}$ (1000 K), $1.15\text{x}10^{18}$ (2000 K) and $1.08\text{x}10^{16}$ $cm^3$ $mol^{-1}$ $s^{-1}$ (4000 K). Center broadening factors $F_{cent}$ were 0.76 (300 K), 0.57 (400 K), 0.26 (800 K), 0.21 (1000 K), 0.12 (2000 K) and 0.12 (4000 K).

## Molecular Parameters

Enthalpy $\Delta H_0^o$ = 541.7 kJ mol$^{-1}$of the dissociation reaction $CF_4 \rightarrow CF_3 + F$ from enthalpies of formation of $\Delta H_{f,0}^o(CF_4)$ = - 927. 20 kJ mol$^{-1}$, $\Delta H_{f,0}^o(CF_3)$ = - 462.8 kJ mol$^{-1}$, and $\Delta H_{f,0}^o(F)$ = 77.29 kJ mol$^{-1}$; data from ref SI4; data from ref SI5 are $\Delta H_{f,0}^o(CF_4)$ = - 927. 15 ± 0.53 kJ mol$^{-1}$, $\Delta H_{f,0}^o(CF_3)$ = - 464.6 ± 1.97 kJ mol$^{-1}$, and $\Delta H_{f,0}^o(F)$ = 77.274 ± 0.058 kJ mol$^{-1}$, leading to $\Delta H_0^o$ = 539.8 kJ mol$^{-1}$.

Vibrational frequencies of $CF_4$ (in cm$^{-1}$): 435.4 (2), 631.1 (3), 909.1, 1283.7 (3) from ref SI6.

Vibrational frequencies of $CF_3$ (in cm$^{-1}$): 508.7 (2), 701.4, 1086, 1253.8 (2) from ref SI7.

Rotational constants of $CF_4$ (in cm$^{-1}$): 0.192 (3) with $\sigma$ = 12; from ref SI8.

Rotational constants of $CF_3$ (in cm$^{-1}$): 0.364 (2), 0.189 with $\sigma$ = 3; from ref SI9.

## References


(SI1) Curtiss, L. A.; Redfern, P. C.; Raghavachari, K. Gaussian–4-Theory. *J. Chem. Phys.* **2007**, *126,* 084108.

(S12) Becke, A. D., Density-Functional Exchange-Energy Approximation with Correct Asymptotic Behaviour. *Phys. Rev. A: At., Mol., Opt. Phys*. **1988**, *38*, 3098−3100.

(SI3) Frisch, M. J. et al., Gaussian 09, revisionA.02 (Gaussian, Inc., Wallingford CT) **2009**.

(SI4) Chemical Kinetics and Photochemical Data for Use in Atmospheric Studies (JPL Publication 19-5. Evaluation number 19, **2020**).

(SI5) Goos, E.; Burcat, A.; Ruscic, B. Extended Third Millenium Ideal Gas and Condensed Phase Thermochemical Database for Combustion with Updates from Active Thermochemical Tables. (http://burcat.technion.ac.il/dir 21 January **2015**).

(SI6) Lolck, J.-E. The Raman Spectrum of $CF_4$. *J. Raman Spectrosc*. **1981**, *11*, 294-301.

(SI7) Forney, D.; Jacox, M. E.; Irikura, K. K. Matrix-Isolation Study of the Interaction of Excited Neon Atoms with $CF_4$. Infrared Spectra of $CF_3^+$ and $CF_3^-$. *J. Chem. Phys*. **1994**, *101,* 8290-8296.

(SI8) Suzuki, T.; Okada, H.; Fujiyama, T. Measurement and Analysis of the $\nu_3$ Band of $CF_4$. *Bull. Chem. Soc. Jpn.* **1979**, *52*, 2505-2511.

(SI9) Endo, Y., Yamada, C., Saito, S., Hirota, E.,The Microwave Spectrum of the Trifluoromethyl Radical, *J. Chem. Phys*. **1982**, *77*, 3376-3382; Yamada, C.; Hirota, E. Infrared Diode Laser Spectroscopy of the $\nu_3$ Band of $CF_3$. *J. Chem. Phys.* **1983**, *78*, 1703-1711.
